# Urban renewal and social sustainability: An exploratory study of the urban life of Wan Chai

**DOI:** 10.1186/2193-1801-4-S2-P5

**Published:** 2015-11-27

**Authors:** Leo Kwun-sing Wong, Peter Hoi-fu Yu

**Affiliations:** Research Support Unit, Vocational Training Council, Hong Kong; Faculty of Science and Technology, Technological and Higher Education Institute of Hong Kong, Hong Kong

## Background

Urban renewal has become an important issue in Hong Kong, which is experiencing considerable population growth pressures and urban decay in various districts. Ho et al. [1] noted that urban renewal often creates not only environmental problems such as demolition waste, but also social problems such as destruction of existing social networks and the fabric of long-established communities. Despite the Urban Renewal Authority (URA)'s claim to foster, encourage, and support sustainable development, it is not difficult to find negative comments from the public about various aspects of urban renewal, in particular with regard to urban sustainability - the social dimension of sustainable development. In fact, in the past ten years, there have been several social movements concerned with or directed against redevelopment programmes in urban areas. What these social movements have in common is that participants are not only affected residents or business owners, but also members of civil society who do not live in the affected districts. Some scholars have started to criticise the URA's planning approach, and stated that there is a need to review the practices of urban renewal in Hong Kong [2].

Through a comprehensive review of urban development, urban planning and urban renewal in Hong Kong, and case studies and observations of Wan Chai, this study attempts to explore the influence of planning ideology and modernist urban planning on urban renewal practices in Hong Kong. It also considers why most residents do not support, and even oppose, the urban renewal projects of the URA.

## Methods

This study makes use of a literature review, case studies and observations. The first case study is of Lee Tung Street, which has a particular significance in terms of cultural heritage preservation because it represents a turning point when the community was united to create a proposal to oppose government planning. Another case study is the Cross Street and Tai Yuen Street open-air bazaar, a traditional street market which is significant in terms of its long history and community-friendly features. This case provides information about local community, street space, and everyday practices.

Complete observation was used in this study. Field observations were carried out on a number of weekdays and on a weekend in June 2013 at a variety of times (from 8:00 am to 9:00 pm). Both Wan Chai North and Wan Chai South were observed, to capture the real situation regarding the different urban structures and people's everyday practices in Wan Chai.

## Results

Based on the two case studies and field observations, the most obvious problem with current urban renewal practice is that it completely fails to consider the way of life of local residents, especially the majority of inhabitants who are elderly and have lived in the old urban areas for decades. Furthermore, it fails to take into account important social values relating to historical and cultural characteristics, and fails to acknowledge the need to consult local communities and other non-governmental actors in the urban renewal process.

## Conclusions

Urban renewal practices in Hong Kong are significantly affected by modernist urban planning approaches. Although urban planners recognise the importance of urban renewal, they focus on the physical environment and do not consider other factors such as residents' everyday lives, social and cultural values, and the most important factor: community. Although it is widely believed that cultural and social diversity is important for developing social capital, it is time for the Government and urban planners to rethink urban planning practices and pay more attention on the everyday lives of communities.
